# Pharmacy Technicians’ Roles and Responsibilities in the Community Pharmacy Sector: A Welsh Perspective

**DOI:** 10.3390/pharmacy8020097

**Published:** 2020-06-04

**Authors:** Rebecca Chamberlain, Jan Huyton, Delyth James

**Affiliations:** 1 Cardiff School of Education and Social Policy, Cardiff Metropolitan University, Cardiff CF5 2YB, UK; jhuyton@cardiffmet.ac.uk; 2 Cardiff School of Sport and Health Sciences, Cardiff Metropolitan University, Cardiff CF5 2YB, UK; dhjames@cardiffmet.ac.uk; 3 Health Education Improvement Wales, Ty Dysgu, Cefn Coed, Nantgarw CF15 7QQ, UK

**Keywords:** pharmacy technician, community pharmacy, roles, responsibilities, barriers, enablers, dispenser, pharmacy services, workforce development, wales

## Abstract

**Background:** Healthcare delivery models in Wales are changing in response to unprecedented pressure on the National Health Service UK (NHS). Community pharmacies will be prioritised to address public health and clinical needs at a local level. To support the delivery of the new model, pharmacy technicians must be enabled and developed to optimize their roles. The aim of the study was to establish existing roles of pharmacy technicians working in the community pharmacy sector in Wales and to explore barriers and enablers to development. **Methods:** A combination of quantitative and qualitative methodologies was used, with the main focus on quantitative methods. A total of 83 participants completed an online questionnaire and additional qualitative data were obtained from four semi-structured telephone interviews. **Results**: The dispensing and final accuracy checking of medicines were reported as core functions of the community pharmacy technician role, with an average of 43% and 57% of time being spent on these roles, respectively. There was some evidence of engagement in leadership and management roles (average of 19%) and limited evidence of delivery of services (average of 6%). **Conclusions:** There is scope to enable community pharmacy technicians to optimize and further develop their roles. Enablers include the effective use of delegation, workplace support, improved staffing levels and the prioritisation of extended pharmacy technician roles.

## 1. Introduction

The National Health Service (NHS) in the United Kingdom (UK) is under immense pressure to deliver quality healthcare with restricted resources [1]. The devolution of healthcare in the UK has led to significant differences in the commissioning of community pharmacy services across the four nations. In Wales, community pharmacies have been identified as a strategic priority for enabling the local delivery of public health and clinical services, with less focus on the supply of medicines [1,2,3]. These proposed changes have the potential to impact on the future roles and responsibilities of the community pharmacy workforce in Wales. In order to achieve the Government’s strategic objectives, it is crucial that the skill mix of the pharmacy team is utilised to its optimum effectiveness [4]. The General Pharmaceutical Council (GPhC) (2018) reported that there were 23,318 pharmacy technicians (PTs) registered in the UK on 31 March 2017, with 53,967 registered pharmacists and 14,403 registered pharmacy premises [5].

PTs made the transition from an occupation to a profession in 2011, following introduction of professional registration—the title ‘Pharmacy Technician’ is protected in UK law [6]. Pharmacy technicians must renew their registration every year, by declaring that they remain fit to practice, in accordance with the GPhC’s professional standards. The registration requirements are mandated by the GPhC and are the same regardless of sector. These are Level 3 National Vocational Qualification (NVQ) Diploma Pharmacy Services Skills (or equivalent), plus a Level 3 Diploma Pharmaceutical Science (or equivalent) and a minimum of 2 years’ relevant work-based experience under the supervision of a pharmacist. Level 3 qualifications are equivalent to A-levels in the UK, a prerequisite to accessing a higher education diploma or degree. Pharmacists in the UK study to reach level 7 (Master’s degree) qualifications in higher education institutions (i.e., universities). Pharmacy technicians in the UK are therefore not qualified at degree level.

PTs play an important role within the delivery of pharmacy services in the UK [7]. Defining the role is inherently difficult as there is no agreed definition or clear demarcation of the boundaries with the other members of the pharmacy team [8,9]. As a general overview, PTs are specialists in the technical aspects of medicines supply, e.g., procurement, stock management, sale, dispensing and final accuracy checking of dispensed medicines. PTs may provide guidance on the use of prescribed medicines and public health advice. PTs may manage technical staff and/or the provision of technical pharmacy services. For comparison, pharmacists are considered specialists in the clinical aspects of medicines supply, e.g., ensuring prescribed medicines are safe and appropriate for patients in terms of dose, form, interactions and contra-indications. Pharmacists are legally accountable for the safe and effective provision and delegation of pharmacy services. Current UK pharmacy legislation requires PTs to work under ‘supervision’ of a pharmacist. The law also recognises that pharmacists have scope to delegate tasks to appropriately trained and competent members of the pharmacy team. Despite the law and professional registration (specifically professional accountability) having been put in place to enable delegation, there appears to have been limited impact on the development of the pharmacy technician role.

The PT role differs significantly across the pharmacy sectors, particularly between community and hospital, which are the two sectors in which most PTs are employed [9]. Anecdotally, PTs working in the community pharmacy sector have traditionally focused on the sale and supply of medicines and related administrative functions. In recent years, accuracy checking of prescriptions has become a more established part of this role [9]. However, there has been limited scope for further development, which could, in part, be due to the lack of career structure and/or progression opportunities [10,11].

Evidence suggests that the PT role in community has remained similar to a ‘dispenser’ or ‘pharmacy assistant’ role [12], which is often limited to dispensing medicines and stock related activities. In general, dispensers or pharmacy assistants do not undertake the final accuracy checking of dispensed medicines, provide advice on prescribed medicines or manage pharmacy services. The terms ‘dispenser’ and ‘pharmacy assistant’ are used interchangeably (the term ‘dispenser’ will be used in this paper), to describe non-registered support staff who are trained to NVQ Level 2 or equivalent. Level 2 qualifications are equivalent to GCSE level education in the UK, which is typically completed in Grade 10 and 11 of high school.

### 1.1. Roles of Pharmacy Technicians

A recent systematic review concluded that PTs who are capable of performing more patient care activities are being underutilised [13]. In 2018, Desselle et al. surveyed 5000 pharmacy technicians across eight states of the United States of America (USA), to establish their involvement with specified practice activities [14]. They reported significant differences between community and hospital roles and a significant involvement with prescription receipt and dispensing. Less involvement was reported for roles such as supervising and checking the work of other technicians, despite participants expressing confidence to undertake such roles. Lower levels of confidence and involvement were reported for clinical roles, e.g., discussing effectiveness of treatment plans and providing medicines related advice. This is consistent with Koehler and Brown’s global online survey of pharmaceutical services in 2017 across 67 countries, where procurement and stock ordering were the most autonomous functions of the PT [15]. John and Brown (2017) also found that the sale and supply of medicines remains the core function of the PT’s role in the UK [9].

A 2016 UK study by Boughen et al. [11] explored PT roles across all sectors, including community pharmacy. Survey responses from 71 community pharmacy technicians (CPTs) described a comprehensive list of roles that were undertaken, which mainly related to sale and supply, with some reference made to extended roles. There was no indication of the proportion of time spent on each of the tasks described, in order to provide an accurate picture of the current role. These findings are useful to inform further research, however, they cannot be generalised, due to limitations in the way the sample was recruited. Boughen et al. concluded that community pharmacy technician roles are less expansive and less clinically oriented than hospital pharmacy technician roles.

In 2016, Bradley et al. [7] surveyed a random sample of 1500 pharmacists and pharmacy technicians in England, to explore perceptions of risk associated with the delegation of duties to support staff carrying out roles without direct pharmacist supervision. Participants categorised twenty-two activities as ‘safe’ (e.g., dispensing), ‘borderline’ (e.g., issuing prescriptions and sales of medicines) or ‘unsafe’ (e.g., clinical activities). When compared with PTs and hospital pharmacists, community pharmacists were found to have a significantly higher perception of risk for the delegation of borderline tasks to support staff and were the least ready for change. This may be a barrier to the full realization of changes in practice and development of the PTs role in a community setting.

To date, there has not been any research to explore CPT roles within Wales, where the Welsh Government is prioritising and investing in the community pharmacy sector, as a mechanism to address localised health needs [16].

### 1.2. Aim

The aim of this study was to establish the existing roles and responsibilities of PTs working in the community pharmacy sector in Wales and to explore potential barriers and enablers to optimal role utilization within the pharmacy team.

## 2. Materials and Methods

### 2.1. Overview of Study

**Figure 1:**
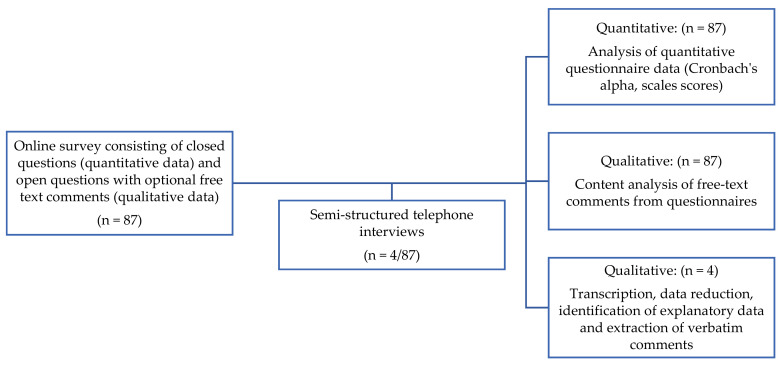
Overview of methodology.

A combination of quantitative and qualitative methodologies was used to address the aim of the study. Whilst the focus of the study was predominantly a quantitative approach, an opportunity was provided for participants to add any comments to the survey and to take part in an in-depth telephone interview, to gather supportive and explanatory qualitative data [17]. See Figure 1. An online questionnaire was sent to PTs across Wales. The questionnaire design was based on the existing literature and extensive professional knowledge of the researcher (RC) and one of the study supervisors (DJ). The questionnaire included mainly closed questions, with the opportunity to provide free text comments. At the end of the questionnaire, participants were offered the opportunity to take part in a short telephone interview, if they wished. PTs were not incentivized to complete the questionnaire or participate in an interview.

### 2.2. Approvals and Ethical Considerations

Ethical approval for this study was obtained from the Research Ethics Committee at Cardiff Metropolitan University’s School of Education and Social Policy (ethic approval code RC0118JH). Attention was paid to informed consent, anonymity, confidentiality, right to withdraw and data protection [18]. The Research and Insight Manager at GPhC approved the questionnaire, before distribution to all registered PTs who were resident in Wales.

### 2.3. Questionnaire

An online questionnaire was developed to gather demographic data, data relating to roles and responsibilities and pre- and post-registration training. Specific data were obtained for workplace support, professional identity and delegation, to ascertain whether or not these were barriers and/ or enablers to current practice and future role development.

Standardised descriptors were used to categorise the type and size of pharmacy [11,12]—independent pharmacy; small chain (2–4 pharmacies); medium sized multiple (5–25 pharmacies); large multiple (over 25 pharmacies); supermarket pharmacy and other. A five-point Likert scale was used to rate agreement with statements relating to perceived barriers and enablers. (SD = strongly disagree; D = disagree; N = neither agree nor disagree; A = agree; SA = strongly agree. NA = not applicable was added where appropriate).

The wording of some Likert scale statements was reversed to reduce the risk of response bias [19]. Free text boxes were included at the end of each set of questions to enable participants to provide further comment and context [20]. The online questionnaire was piloted using three volunteers within the researcher’s [RC] professional network and one of the supervisors (DJ), with expertise in questionnaire design. Revisions were made to improve the clarity of supporting information, the accuracy of rating scales and to resolve technical issues with how the survey was displayed (e.g., when responses to question 7 were filtered through to questions 8 and 9).

### 2.4. Interviews

Semi-structured, one-to-one telephone interviews were undertaken to obtain further insight into community PT roles and the barriers and enablers experienced. Participants were sent a participant information sheet and provided written consent for the interview to be recorded. A semi-structured interview schedule was developed, based on the existing literature and in-depth knowledge of two authors (RC and DJ) of the pharmacy technician workforce. This was used to identify the main topics for discussion and ensure consistency. Open-ended questions and prompts based on potential responses were prepared in advance to provide structure, whilst retaining some flexibility and allowing participants to determine the level of detail provided [21] (see Supplementary Materials). Interviewees were asked about their current roles, use of knowledge and skills in the workplace, efficacy of initial education and training and any further training undertaken. The interview also explored workplace support, delegation and potential enablers and barriers to conducting their role. Interviews were audio recorded and transcribed verbatim.

### 2.5. Sampling, Recruitment and Study Procedures

The GPhC register data do not differentiate registrants by sector of pharmacy, so a specific sampling framework could not be identified. The number of CPTs in Wales was estimated based on data indicating that 6.8% of all PTs in the UK live in Wales [22], equivalent to 1586. Of those, it has been estimated that 53% of PTs work in the community pharmacy sector [23], which is equivalent to 841.

The online questionnaire was disseminated to all registered pharmacy technicians in Wales by the GPhC in January 2018 via e-mail. The launch of the questionnaire was advertised via multiple pharmacy related social media platforms, clearly stating that the questionnaire was intended for community pharmacy technicians only. An initial filter question was added to the questionnaire to avoid completion by non-community-based PTs (32 responders who were not CPTs were redirected to the end of the questionnaire). The GPhC sent two follow up e-mails in February and March, both of which increased response rates.

The questionnaire was hosted on Qualtrics ^©^ software. An open access web link was added to the e-mail message and social media posts. Responses were captured over a 2-month period between January and March 2018. Interview audio data were transcribed by Sterling Transcription^©^, using Intelligent Verbatim (Standard Style).

### 2.6. Data Analysis

Quantitative analysis was undertaken using the report function within Qualtrics software (Version 2018 of Qualtrics, Copyright^©^ 2018 Qualtrics, Provo, UT, USA); e.g., to calculate frequency distribution of demographic and categorical data. Data were extracted from Qualtrics into Microsoft Excel (Excel 97–2004, Microsoft Corporation, Redmond, WA, USA) to calculate central tendencies for interval data; e.g., percentage time spent dispensing per week. Data were also extracted from Qualtrics to the Statistical Package for the Social Sciences (SPSS Version 25.0 2018, IBM Corp, Armonk, NY, USA). Cronbach’s alpha analysis was undertaken to estimate the internal consistency (reliability) of the scales. Negatively worded items were reversed scored and items which contributed to a poor alpha score were excluded from the respective scale (i.e., Q31R etc.) Scales with Cronbach’s alpha scores > 0.7 were deemed to have good internal reliability [23] and therefore total scale scores were calculated for the following scales: efficacy of initial education (5 items), colleagues’ understanding of training (2 items), workplace support (3 items), professional identity (3 items) and delegation (2 items). A Kruskal–Wallis test was used to compare responses from PTs across different categories of pharmacy, for questions relating to workplace support, professional support, recognition of professional identity and use of delegation.

Qualitative content analysis was undertaken by manually coding free-text written comments from questionnaires into categories, e.g., specific job roles, with one exception where quantitative content analysis was undertaken to measure the number of participants who reported a change in role since qualifying as a pharmacy technician. Categories were then grouped together to identify key themes, e.g., areas of pharmacy practice. Interview data were transcribed and simplified using a process known as data reduction [24], to produce a chart summarizing responses to each of the research topics and to identify further explanatory or supporting data. Verbatim quotes were extracted from the interview data for illustrative purposes. Quotes include the participant number, category of pharmacy and year of qualification for context.

## 3. Results

### 3.1. Participant Characteristics

A total of 83 questionnaires were fully completed, which represented approximately 10% of the PT population in Wales. Participant characteristics are presented in Table 1.

The number of years which participants had been qualified varied considerably from 1 year to 38 years, with an average of 13 years. The majority of participants (69%) qualified prior to the introduction of professional registration in 2011. The majority of participants (89%) had worked as a dispenser prior to becoming a PT.

All four participants who indicated their consent for a follow-up interview were contacted by telephone. Two male and two female participants were interviewed. Interviews lasted 29 to 52 min, with an average of 37 min. As the purpose of the interviews was to provide further explanation, relevant summaries of the interview data and verbatim quotes are presented alongside the questionnaire results.

**Figure 2:**
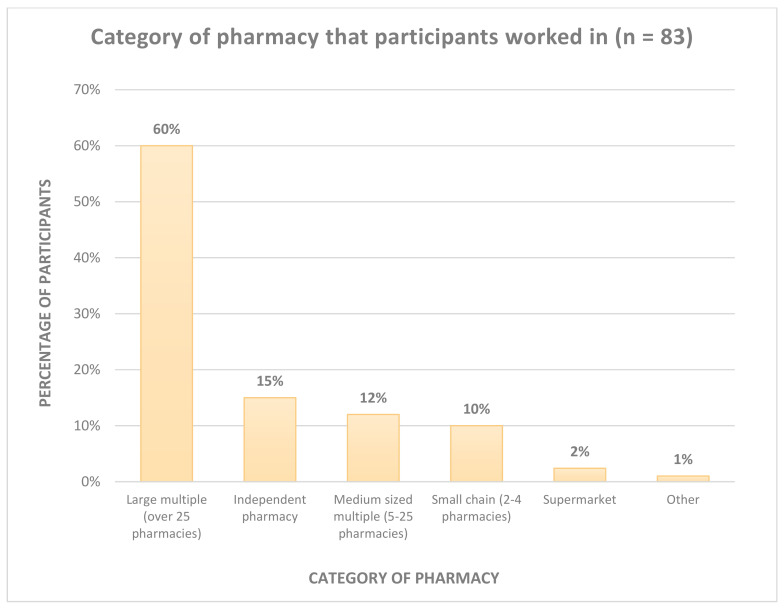
Category of pharmacy in which participants worked.

Figure 2 displays the type of community pharmacy in which the participants worked. The majority of participants worked in a large multiple pharmacy (60%).

**Figure 3:**
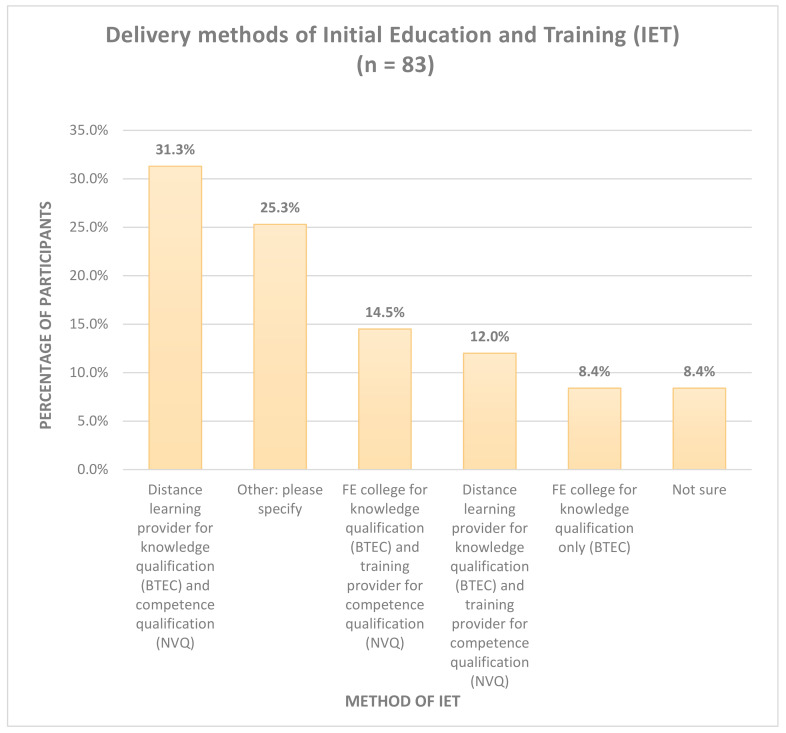
Delivery methods for Initial Education and Training (IET). Key: BTEC = Business and Technology Education Council; NVQ = National Vocational Qualification; FE = Further Education.

Figure 3 illustrates the delivery method of initial education and training (IET). The majority of participants studied via distance learning for BTEC and NVQ qualifications. A quarter (25.3%) of participants selected ‘other’—where there were multiple references to studying via distance learning.

**Figure 4:**
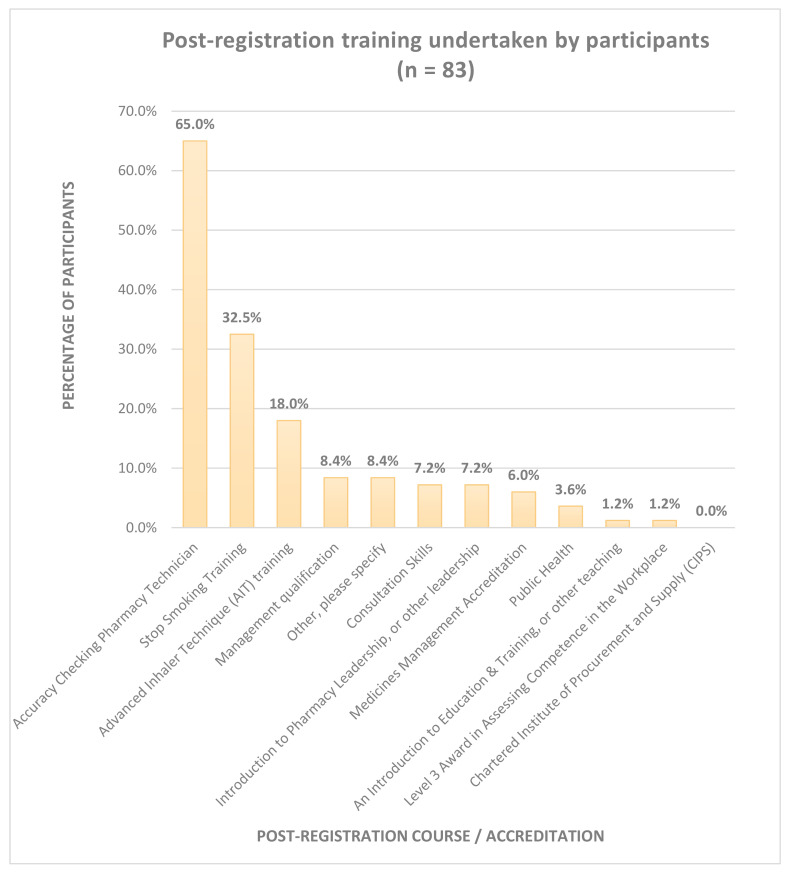
Post Registration training undertaken by participants.

Figure 4 presents the post-registration training undertaken by participants. The total number of responses exceeds 83, as participants were able to select as many options as applied. Two thirds (*n* = 54) of CPTs were trained as accuracy checkers and a third (*n* = 27) had undergone stop smoking training. A fifth of participants (*n* = 15) had received advanced inhaler technique (AIT) training.

Of the 54 trained accuracy checking pharmacy technicians (ACPTs), 26 reported that it had enabled them to final accuracy check prescriptions. Of the 27 stop smoking trained participants, 4 reported that they were enabled to deliver smoking cessation services. Of the 15 AIT trained participants, four reported that they were enabled to deliver smoking cessation services. Two of these took part in the semi-structured interviews. Interviewee B (independent pharmacy) reported,

The smoking [training] certainly did [enable me to undertake the role effectively], because we’ve got a lot of patients on the program, so we do a lot of that in our area, which is good. I—it was just myself and the two pharmacists that—in our two stores and my—the pharmacist here is usually quite busy with other things. So, I tend to take the smoking cessation patients, which is fine with me, because I quite like the—being able to do that service, so—and the training was quite extensive and really in-depth. So, I was able to take that up from day one.

In contrast, Interviewee A (large multiple) stated,

“Well with the independent [previous employer] I did the smoking with him, did that extra training, so I was certified for that”.

Interviewee A wasn’t involved in the smoking cessation service at that time of the interview and went on to explain,

It’s lapsed. It’s lapsed, I just haven’t used it for so long working with the company I work for now, they just—you know most of the pharmacists kind of deal with that, so there are technicians that do do it in the company, but as for me it just never happened.

### 3.2. PT Roles

**Figure 5:**
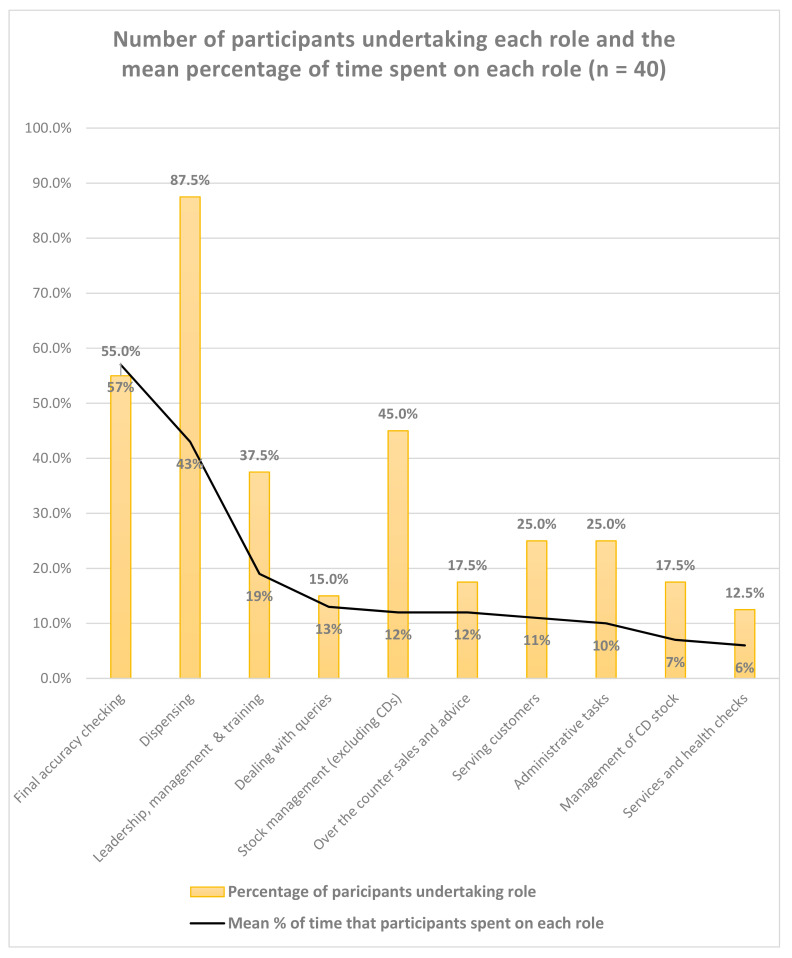
Number of participants and % of time spent undertaking each role.

Qualitative free text comments describing current roles were categorized to identify core roles. Participants were asked to assign a percentage of time to each role they described—only 40 responses were correctly recorded (i.e., percentages were recorded and totalled 100%). Figure 5 is based on 40 (48%) responders, which shows that the dispensing of medicines remains a core role for CPTs. The data further highlight that CPTs who final accuracy check and spend over half their time engaged in the checking role. The data shows that few CPTs are working in leadership, management and/or training roles, and those who are, spend less than 20% of their time engaged in the role.

The interview data further support the above findings. Interviewee A, C and D’s roles related mainly to the sale and supply of medicines, e.g., dispensing and stock management. Interviewee A also described a limited supervisory role, e.g., training new staff and overseeing workload when locum pharmacists are present, and Interviewee C undertook blood pressure checks periodically. In contrast, Interviewee B’s role was split between final accuracy checking and supporting delivery of enhanced services, e.g., targeting appropriate patients and administration of the Medicines Use Review (MUR) service. Interviewee B also reported that they were accredited to deliver the Level 3 Smoking Cessation and appeared more involved in the professional aspects of this service.

Participants were asked whether they had previously worked as a dispenser, where 74 (89%) reported that they had. Of those, 64 participants provided further comments, where 13 (20%) reported little or no difference between the two roles, 3 (5%) reported no difference other than the final accuracy checking role and 48 (75%) described important differences. Differences mainly related to a change in level of responsibility, final accuracy checking role, greater knowledge to provide advice and deal with queries, involvement in training, leadership and management and more respect and value for the role.

More responsibility and more respected as a team leader.

(P83, large multiple, 2017)

As a PT have the knowledge to answer questions / queries from customers with confidence.

(P75, large multiple, 2005)

The pharmacist starting delegating more responsible roles to me. The knowledge I gained was used more effectively and I was allowed to demonstrate how my competence had improved. I felt I was trusted with more responsibility, because I worked in a more professional manner.

(P53, medium sized multiple, 2003)

More responsibility—more involvement in problem solving.

(P40, small chain, 2004)

### 3.3. Perceived Barriers and Enablers

Table 2 summarises the number of items, Cronbach Alpha, ranges, mid-points and scores for each of the five scales within the questionnaire. Each scale is illustrated and discussed further below.

**Figure 6:**
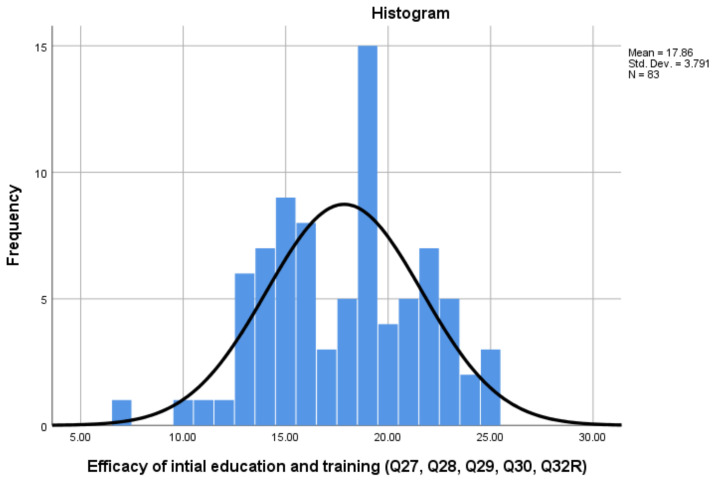
‘Efficacy of Initial Education and Training’ scale scores.

Figure 6 presents participants’ views about the efficacy of their initial education and training (Q27, 28, 29, 30 and 32R). The Cronbach alpha for the ‘Efficacy of Initial Education and Training’ scale was 0.775, with scores ranging from a minimum of 7 to a maximum of 25. The results indicate that two thirds of participants felt their initial training had enabled sufficient development of the knowledge and skills required of the pharmacy technician role, with 68.7% scoring above the mid-point scale score of 15.

**Figure 7:**
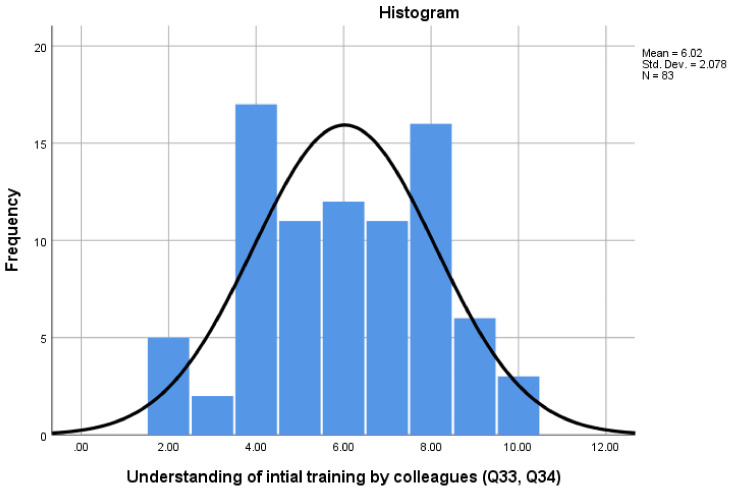
’Colleague Understanding of IET’ scale scores.

Figure 7 presents participants’ views on their colleagues’ understanding of IET (Q33 and Q34). The Cronbach alpha for the ‘Colleague Understanding of IET’ scale was 0.703, with scores ranging from a minimum of 2 to a maximum of 10. The results show a wide range in scores, which suggests that there may be a lack of understanding around the IET curriculum and the role of a pre-registration pharmacy technician, with 43.4% scoring at or above the midpoint scale of 6.

Participants were invited to make additional comments about their IET. Twenty-three participants provided qualitative comments and four themes were identified; the need to improve the relevance of IET and opportunities to apply it; the importance of experiential learning to develop skills; the need for workplace support and the challenges of learning in the workplace.

“Whilst interesting most of what I learnt for the NVQ 3 has never been used in my current position”.

(P15, large multiple, 2002)

“The training was a base for a pharmacy tech, the skills needed are learnt through experience, it’s not an easy job to do and definitely needs in depth training to fully cover all aspects of the job role”.

(P49, large multiple, 2011)

“Would have liked an on-site visit to assess my work, found the assessment was not portraying my work, instead of paperwork through my course”.

(P46, independent pharmacy, 2012)

**Figure 8:**
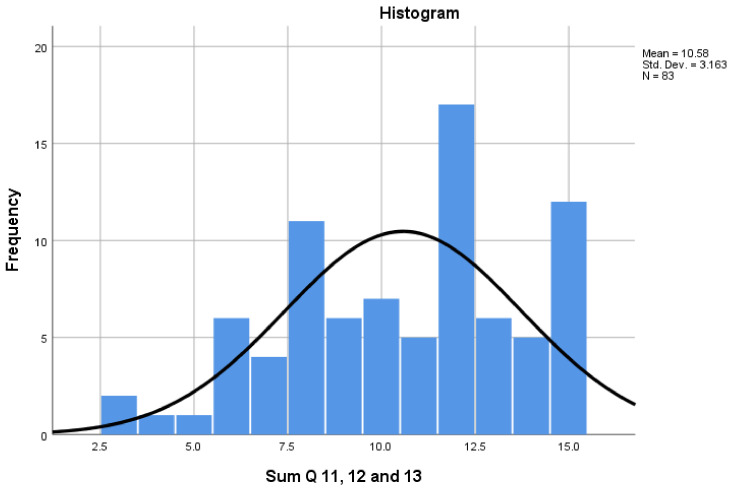
‘Workplace Support’ scale scores.

Figure 8 presents participants’ views about workplace support (Q11, Q12 and Q13). The Cronbach alpha for the ‘Workplace Support’ scale was 0.663, with scores ranging from a minimum of 3 to a maximum of 17. The results suggest that two thirds of participants felt supported in the workplace, with 72.7% scoring above the mid-point of 9. The results also indicated that CPTs receive most support from pharmacist colleagues and that a quarter (*n* = 23) of CPTs do not have PT colleagues in their workplace.

**Figure 9:**
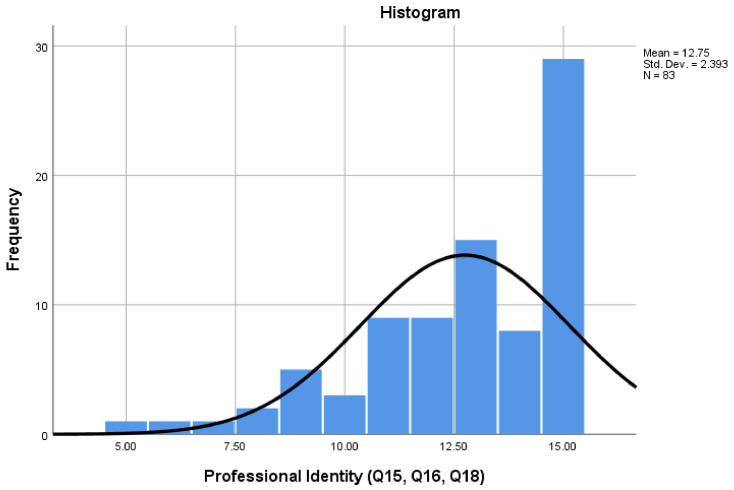
‘Professional Identity’ scale scores.

Figure 9 reports participants’ views on professional identity (Q15, Q16 and Q18). The Cronbach alpha for ‘Professional Identity’ was 0.700, with scores ranging from a minimum of 5 to a maximum of 15. The results suggest that the majority of CPTs have adopted a professional identity, with 88% scoring above the mid-point score of 9.

**Figure 10:**
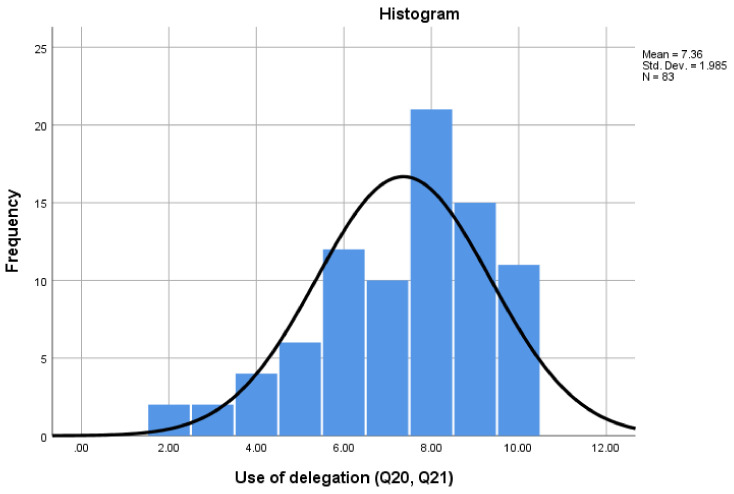
‘Delegation’ scale data.

Figure 10 illustrates participants’ views on delegation in their workplace (Q20 and Q21). The Cronbach alpha for ‘Delegation’ was 0.666, with scores ranging from a minimum of 2 to a maximum of 10. The results suggest that although delegation is utilised there, there could be scope to utilise it more effectively, with 68.7% scoring at or above the mid-point score of 6. This is consistent with interview data, in which Interviewees A and D reported that only pharmacists or management staff delegate work, whereas Interviewees B and C reported that they could delegate unscheduled tasks. Interviewees C and D reported that Dispensers and PTs undertook similar tasks, though Interviewee C stated that the dispenser refers any issues to a PT. Interviewee A reported that the PT role was intended to focus on running the pharmacy, but in practice, they often ended up covering dispensers’ role, e.g., retail sales. Interviewee B (independent pharmacy) stated,

So the ACT dispense a lot less that what they used to, because we have people who dispense and then the ACTs do the checking. We—there’s a lot more of a defined role now. I mean, I think we could still be better and there’s still a lot we could do, but I think we’re definitely moving in the right direction.

Participants were asked to describe factors which enabled them to undertake their role effectively. Fifty-three (64%) of participants provided qualitative responses and the main theme which emerged was support, i.e., team working, pharmacist support and managerial support. Adequate and on-going training was also highlighted.

Support of the superintendent pharmacist which enables me to lead and develop my team.

(P6, independent pharmacy, 2009)

A strong supportive non-pharmacy manager plays a huge role in my pharmacy. Colleagues who work together in a very busy pharmacy.

(P10, medium sized multiple, 2015)

My pharmacy manager gives me the encouragement and confidence for me to undertake my role effectively.

(P38, large multiple, 2016)

Quality of training. The right person in the right task. Clean and efficient working environment. Good managerial team.

(P66, large multiple, 2006)

I have continual trading [training] from my pharmacist to ensure I’m up to date with what I’m checking.

(P73, medium size multiple, 2001)

The interview data provided a contrasting picture, with all four interviewees reporting limited workplace support. Interviewees C and D suggested they accessed support from specific pharmacist colleagues with whom they had a good relationship, whereas Interviewees A and B suggested that the only guidance or support received was from SOPs and annual performance reviews, respectively.

Participants were also asked to describe factors which were barriers to them undertaking their roles effectively. Fifty-nine (71%) participants provided qualitative responses and two key themes were identified. The first was staffing issues, i.e., inadequate staffing and lack of qualified or competent staff. The second was business pressures, i.e., busy environment, insufficient time and the prioritisation of targets.

We don’t have enough staff for me to do my job role. I am the only qualified technician in our dispensary.

(P7, large multiple, 2016)

Enough staff to have more time to take on the roles I would like to do.

(P36, large multiple, 2003)

The pressure of a very busy pharmacy sometimes means you don’t have enough time to interact with patients.

(P9, large multiple, 1993)

Pharmacists having to take on too many services. Not enough time for them to do any prescription checking.

(P58, large multiple, 2002)

Finally, participants were asked to identify any roles which they felt confident to do but did not currently undertake. From the thirty-one qualitative responses, four key themes emerged; enhanced services (e.g., weight loss, stop smoking and medicines usage review); extended roles (e.g., final accuracy checking); training and development and counselling and advice. The reasons given for non-engagement in these roles included; lack of relevant training, lack of time, staff shortages, minimal pay increase, domain of the pharmacist, demands of repeat dispensing, automation of dispensing process, lapsed accreditation and health board restrictions.

Offering weight loss service and stop smoking device [service] in pharmacy, unable to at present as not undertaken relevant training yet.

(P77, independent pharmacy, 2013)

More enhanced services, specifically DMR (discharge) and flu jabs. I see no reason why fully qualified technicians can’t learn and provide the service. Also we should be carrying out MURs in the home to the patients who need more assistance. Technicians should somehow be able to assist with that and be able to do the home visits and the medicines management.

(P60, independent pharmacy, 2008)

There are lots of service roles that are aimed at pharmacists—no smoking, weight control etc. that both technicians or pharmacists could do but both are hampered by the continuous and increasing demands or repeat dispensing.

(P56, independent pharmacy, 2009)

Checking prescriptions which I can no longer do due to the introduction on [of] advance dispensing and robot dispensing.

(P55, large multiple, 2002)

Mentor staff when doing courses. I do this but only in a casual way. The pharmacist does this officially.

(P45, independent pharmacy, 2001)

### 3.4. Relationship between Type of Pharmacy and Barriers and Enablers

There were no statistically significant differences between the category of pharmacy and the median responses to the sets of questions relating to efficacy of IET (H (df = 5) = 4.249, *p* = 0.514), colleague understanding of IET (H (df = 5) = 6.645, *p* = 0.248), workplace support (H (df = 5) = 6.751, *p* = 0.240), professional identity (H (df = 5) = 7.514, *p* = 0.185) and delegation (H (df = 5) = 4.410, *p* = 0.492).

## 4. Discussion

Survey responses provided data from across all categories of pharmacy and from CPTs, with varying educational backgrounds. The GPhC ‘Survey of registered pharmacy professionals 2019’ [25] provides data to suggest that the sample within this research could be considered representative of CPTs in Wales. The GPhC reported that 6% of the 23,506 registered PTs were resident in Wales (1410) and that 56% of PTs in Wales work in community pharmacy. This suggests there were 789 CPTs in Wales in 2019, which is consistent with the estimated sample size of 841 in 2018. The 2019 survey showed that 64% of CPTs in Wales accessed the register via the grandparent route, i.e., prior to 2011, which is comparable with the research sample in which over two-thirds of CPTs qualified prior to 2011. The average number of hours worked per week by CPTs in Wales in 2019 was reported as 32.4, which is broadly equivalent to the average reported in this study. The 2019 survey showed that 55% of CPTs in Wales worked in a large multiple and 20% in a small to medium chain; again, this is consistent with our findings, where over half worked in a large community pharmacy and a fifth worked in a small or medium sized chain. Finally, 57% of PTs across all sectors in Wales reported that they held an ACPT qualification—which was slightly higher (two-thirds) in our study sample. It is therefore reasonable to conclude that the research sample is representative of CPTs in Wales and therefore the results can be generalised to the wider population of CPTs in Wales.

The aim of this study was to explore the roles and responsibilities of CPTs in Wales and identify potential barriers and enablers to role development. In summary, the dispensing of medicines remains a core role for CPTs in Wales, despite there being opportunity to delegate the role to appropriately trained, non-registered support staff, which make up 64% full time equivalent (FTE) roles in community pharmacies in Wales [26]. This finding is consistent with Salameh et al.’s (2018) [27] exploratory study, in which all 16 PT participants reported dispensing as a day-to-day responsibility, and the recent 2019 GPhC survey [25], in which 85% of CPTs in Wales reported supplying medicines and medical devices as a main role. Failure to enable delegation of the dispensing process and fully utilise the skill mix within the pharmacy team, is a barrier to CPTs in Wales fulfilling their potential, even within their existing roles.

The results also suggest that final accuracy checking is becoming a core role and that ACPTs spend approximately half their time final accuracy checking. However, the data also suggest that not all trained ACPTs are being enabled to final accuracy check, often due to capacity issues. This finding is consistent with a recent workforce survey [26], which concluded that “the required opportunities and infrastructure should be made available to increase the percentage of community pharmacy technicians accredited to accuracy check prescriptions to match hospital levels over the next 3 years”. Similarly, the data suggest that there is limited engagement in service-based roles, e.g., smoking cessation and inhaler techniques counselling, even when CPTs have completed the required training and there are data suggesting CPTs are willing to undertake these roles. These findings are consistent with Doucette and Schommer’s [28] survey research, which found that insufficient staffing levels, insufficient time and lack of employer recognition for specialized skills, were barriers to PTs engaging in emerging tasks. These findings support the need for the community pharmacy sector in Wales to urgently address the capacity issues which are a current barrier to CPTs engaging in roles which they are trained and/or are willing to be trained to undertake. Taking these measures would support the Welsh Government’s vision of localised delivery of public health and clinical services.

There is evidence of an explicit career pathway, from Dispenser to PT in the community pharmacy sector in Wales. In 2018, the Welsh Government announced a commitment to support the education of up to two hundred PRPTs over three years and community pharmacy contractors were invited to nominate suitable candidates—it is likely that this funding will continue to support the development of dispensers to PTs [29]. The data suggest that many PTs recognise important differences between the two roles in terms of responsibility, knowledge required and respect or value for the PT role—this is a marked divergence from existing research [12,27]. The majority of participants previously worked as dispensers, and despite this, they appear to have transitioned and adopted a professional identity. Salameh et al. (2018) [27] found that professional identity formation was one of four key areas required to optimise the PT role in the community sector, hence these results are encouraging in terms of laying the foundations for future role development.

The existing IET appears to be sufficient to support the foundation PT role, with the majority of PTs reporting that their initial training enabled them to undertake their role on day one. IET could be further improved by ensuring the curriculum accurately reflects the PT role, placing a greater emphasis on experiential learning and improving workplace support for PRPTs. What appears to be more of a barrier is the lack of understanding of the IET and/or the role of a pre-registration pharmacy technician (PRPT) by colleagues. This could be related to the use of distance learning courses, where PRPT training is often facilitated by a single pharmacist who may review work and/or act as an expert witness, but where summative assessment decisions are made by external assessors employed by the education provider [12]. The new combined qualification which is currently being introduced [30], must meet the GPhC (2017) revised standards for IET [31], which state that systems must enable PRPTs to meet regularly with colleagues to review and document their progress. Similarly, the GPhC ‘Guidance on tutoring and supervising pharmacy professionals in training’ [32] explicitly states that a designated educational supervisor must have oversight of training and assessment in the workplace, overall responsibility for supervision and sign the final supervisory declaration. The new qualification standards allow PRPT training to be supervised by a PT, not just a pharmacist, as was previously the case. It is hoped that the new standards will improve colleague engagement and understanding of the IET curriculum and the PRPT role, which could lead to increased confidence in PT competencies and facilitate more informed decision making around the use of delegation and potential PT roles.

Despite some conflicts within the data, it does appear that workplace support is an important enabler for CPTs, particularly support from pharmacists and managers. The support provided appears to be informal in nature, e.g., encouragement and confidence building, and often provided by colleagues with whom the CPT has a good working relationship. Whilst this could be sufficient to support CPTs within their existing roles, CPTs would benefit from access to more formal support in the workplace, such as mentoring or peer support, to enable them to further develop their roles with confidence.

This study has several limitations. The questionnaire data did not include the participants’ age or gender—the authors acknowledge that gender could be considered a relevant factor, as 90% of the PT profession are female [22], whilst noting that the gender of the interviewees was balanced. The quality of data could have been affected by recall bias, when participants were asked to report on their practice as a ‘day one’ pharmacy technician and their career since. It may also have been affected by the willingness of participants to report on some topics, e.g., being open about the barriers experienced [33]. The validity of the survey data could have been affected by non-response bias, though the authors note that the 2019 GPhC study [25] yielded a 25% response rate for PTs in Wales across all sectors (not just community sector), and this highlights the difficulty in reaching this population. Although the response rate is low, this is consistent with those of similar studies with PTs. However, the authors acknowledge that care should be taken when generalising these findings. Due to the limited volume of research into PT roles in Wales, or indeed the UK, there was little opportunity to use previously validated questions. The wording of one question, ‘Which role/s did your further training enable you to undertake’ was potentially ambiguous. The word ‘enable’ could have been interpreted as competence and/or confidence to undertake the role, or as opportunities to undertake the role within the workplace. The limitations of Likert scales include the assumption that subjective data can be quantified and that intervals on a Likert scale are equally spaced. The issue of quantifying subjective data was addressed to some degree, by the inclusion of free text boxes, to enable participants to provide further context [20]. The questionnaire was purposely designed to enable participants to describe their role in their own words, rather than compelling participants to select roles from a pre-determined list. However, the use of open-ended questions is a known factor in survey fatigue [34] and may have affected completion rates. The questionnaire was also designed to quantify the amount of time participants spent undertaking each role, to identify core roles and responsibilities. Unfortunately, under half of respondents completed this section correctly. Some participants simply did not assign a percentage to each role, whereas other participants assigned percentages which did not add up to 100%. The erroneous responses had to be omitted from the analysis of this section, which reduced the reliability of the data.

The authors recognise that the validity of the interview data may be compromised by the low response rate and note that time constraints did not allow for further recruitment of participants. Telephone interviews present specific challenges for researchers; e.g., the sample of participants who are accessible via telephone may not be representative, it may be more challenging to develop a rapport with participants over the telephone [35], participant responses may be affected by the perceived anonymity that distance provides, researchers cannot use visual aids and neither party has access to non-verbal language and cues [36].

The lead author and interviewer (RC) is a PT and acknowledged that their experiential knowledge of the profession shaped their approach to the research, e.g., the barriers and enablers explored. RC also considered the potential impact of ‘role power’ and was careful to differentiate the research from other employed roles. To avoid a one-way discourse during the interviews, a semi-structured interview format was favoured.

An alternative approach to undertaking this research may have been to observe CPTs in the workplace, or to conduct more in-depth interviews to establish core roles. This approach would also have enabled further exploration of how professional identities are developed, which was beyond the scope of this study, but could highlight another important area for further qualitative research. The scope of this study was limited by time and resources; however, it is recognised that future research would benefit from the inclusion of pharmacist perspectives, particularly around the issues of delegation and efficacy of IET.

This study has been circulated internally at the General Pharmaceutical Council (GPhC) to individuals working in education, policy, revalidation, communications and insight, intelligence and inspection. At the time of writing, the outcomes of this are as yet unknown. The study has also been shared with the Pharmacy Dean at Health Education and Improvement Wales (HEIW) and the Chief Pharmaceutical Officer for Wales. The study has been referenced within HEIW’s Wales Community Pharmacy Workforce Survey 2019 [26].

## 5. Recommendations and Conclusions

The findings of this study indicate that CPTs’ knowledge and skills are not being utilised to the full extent even within existing roles. There is also evidence to suggest that CPTs are willing and able to undertake extended roles such as smoking cessation services, if they are enabled to do so. If the Welsh Government’s vision for community pharmacy services is to be fully realised, the existing potential of the PT workforce within Wales must be recognised and the further development of PT roles must be prioritised. Whilst the ability to make firm conclusions is limited by the small response rate, there are a number of recommendations that could be taken forward, based on these findings. These are:

1. Community pharmacy employers and stakeholders should recognise the potential of the CPT workforce and address the barriers to optimisation of the current CPT role in Wales.
2. Community pharmacy employers and stakeholders should prioritise the training and development of CPTs and enable them to undertake extended roles, which will support the future delivery of pharmacy services in Wales.
3. Following the introduction of the new IET standards and guidance on tutoring, the GPhC should explore the impact of the standards and guidance on the training experiences of PRPTs and the understanding of the IET requirements by the wider pharmacy team.
4. Further qualitative research into the CPT workforce in Wales should include further exploration of time spent on specific roles, and the exploration of how professional identities are developed, as well as the views of pharmacists on issues such as the delegation and efficacy of IET.

## Figures and Tables

**Table 1:** Participant Characteristics (*n* = 83).

Characteristic	Frequencies
Number of years qualified	Range 1 to 38; Mean = 13 (SD = 8.2)
Qualified pre mandatory registration in 2011	*n* = 60 (69%)
Worked as a Dispenser prior to becoming a PT	*n* = 74 (89%)
Number of hours worked a week	Range 12 to 45; Mean 31.7 (SD = 7.84)

**Table 2:** Summary of scale scores.

Scale	Number of Items	Cronbach’s Alpha	Scale Range	Scale Mid-Point	% above Scale Mid-Point
Efficacy of Initial Training	5	0.775	7–25	15	68.7%
(scale 5 to 25)					
Colleagues Understanding of IET	2	0.703	2–10	6	43.4%
(scale 2–10)					
Workplace Support	3	0.663	3–15	9	72.7%
(scale 3–15)					
Professional Identity	3	0.700	5–15	9	88.0%
(scale 3–15)					
Delegation	2	0.666	2–10	6	68.7.%
(scale 2–10)					

## Supplementary Materials

The following are available online at https://www.mdpi.com/2226-4787/8/2/97/s1, Participant Interview Schedule.

## References

[1] Welsh Government (2018). A Healthier Wales: Our Plan for Health and Social Care. https://www.basw.co.uk/system/files/resources/180608healthier-wales-mainen.pdf.

[2] Welsh Government (2017). Written Statement Community Pharmacy Funding 2017-18 and beyond. https://gov.wales/written-statement-community-pharmacy-funding-2017-18-and-beyond.

[3] Robinson J. (2017). Diverging community pharmacy practice across the four UK nations. Pharm J..

[4] Welsh Government (2014). Written Statement Delivering Prudent Healthcare within Wales. https://gov.wales/written-statement-delivering-prudent-healthcare-wales.

[5] General Pharmaceutical Council (2018). Annual Report 2016-2017. https://www.pharmacyregulation.org/sites/default/files/gphc_annual_report_2016-17.pdf.

[6] General Pharmaceutical Council (2013). Criteria for Registration as a Pharmacy Technician. https://www.pharmacyregulation.org/sites/default/files/Registration%20criteria%20for%20pharmacy%20technicians%20May%202013.pdf.

[7] Bradley F., Willia S.C., Noyce P.R., Schafheutle E.I. (2016). Restructuring supervision and reconfiguration of skill mix in community pharmacy: Classification of perceived safety and risk. Res. Soc. Admin. Pharm..

[8] General Pharmaceutical Council (2015). Tomorrow’s Pharmacy Team, Future Standards for the Initial Education and Training of Pharmacists, Pharmacy Technicians and Pharmacy Support Staff. https://www.pharmacyregulation.org/sites/default/files/tomorrows_pharmacy_team_june_2015.pdf.

[9] John C., Brown A. (2017). Technicians and other pharmacy support workforce cadres working with pharmacists: United Kingdom Case Study. Res. Soc. Admin. Pharm..

[10] Howe H., Wilson K. (2012). (2012) Modernising Pharmacy Careers Programme. Review of Post-Registration Career Development of Pharmacists and Pharmacy Technicians, Background Pape. http://docplayer.net/3056835-%C2%AD%E2%80%90Modernising-%C2%AD%E2%80%90pharmacy-%C2%AD%E2%80%90careers-%C2%AD%E2%80%90programme-%C2%AD%E2%80%90review-%C2%AD%E2%80%90of-%C2%AD%E2%80%90post-%C2%AD%E2%80%90registration-%C2%AD%E2%80%90career-%C2%AD%E2%80%90development-%C2%AD%E2%80%90of-%C2%AD%E2%80%90pharmacists-%C2%AD%E2%80%90and-%C2%AD%E2%80%90pharmacy-%C2%AD%E2%80%90technicians.html.

[11] Boughen M., Fenn T., Croot J., Frost K., Family H., Wright D., Sutton J. Identifying the Roles of Pharmacy Technicians in the UK, Final Report, September 2016. https://www.uea.ac.uk/documents/899297/15294873/Identifying+The+Role+Of+Pharmacy+Technicians+In+The+UK/d6d60e7b-f527-481a-8f16-9f3f04037b6c.

[12] Schafheutle E.I., Jee S.D., Willis S.C. (2018). The influence of learning environments on trainee pharmacy technicians’ education and training experiences. Res. Soc. Admin. Pharm..

[13] Mattingly A.N., Mattingly T.J. (2018). Advancing the role of the pharmacy technician: A systematic review. JAPhA.

[14] Desselle S.P., Hoh R., Holmes E.R., Gill A., Zamora L. (2018). Pharmacy Technician self-efficacies: Insight to aid future education, staff development, and workforce planning. Res. Soc. Admin. Pharm..

[15] Koehler T., Brown A. (2017). A global picture of pharmacy technician and other pharmacy support workforce cadres. Res. Soc. Admin. Pharm..

[16] Welsh Government (2019). A Healthier Wales: Our Plan for Health and Social Care. https://gov.wales/sites/default/files/publications/2019-10/a-healthier-wales-action-plan.pdf.

[17] Bryman A. (2016). Soc. Research Methods.

[18] British Educational Research Association Ethical Guidelines for Educational Research. https://www.bera.ac.uk/wp-content/uploads/2014/02/BERA-Ethical-Guidelines-2011.pdf?noredirect=1.

[19] Bowling A., Bowling A., Ebrahim S. (2005). Quantitative Social Science: The survey. Handbook of Health Research Methods.

[20] Rattray J., Jones M.C. (2007). Essential elements of questionnaire design and development. J. Clin. Nurse.

[21] Miller R.L., Brewer J.D. (2007). The A-Z of Soc. Research.

[22] Seston L., Hassell K. Briefing Paper: GPhC Pharmacy Technician Register Analysis 2012. https://www.pharmacyregulation.org/sites/default/files/document/gphc-pharmacy-technician-register-analysis-2012.pdf.

[23] General Pharmaceutical Council (2014). Registrant Survey 2013, Initial Analysis. https://www.pharmacyregulation.org/sites/default/files/gphc_registrant_survey_2013_initial_analysis.pdf.

[24] Cohen L., Manion L., Morrison K. (2011). Research Methods in Education.

[25] General Pharmaceutical Council Survey of Registered Pharmacy Professionals 2019. https://www.pharmacyregulation.org/sites/default/files/document/gphc-2019-survey-pharmacy-professionals-main-report-2019.pdf.

[26] Health Education and Improvement Wales (HEIW) (2020) Wales Community Workforce Survey Report 2019.

[27] Salameh L., Yeung D., Surkic N., Gregory P., Austin Z. (2018). Facilitating integration of regulated pharmacy technicians into community pharmacy practice in Ontario: Results of an exploratory study. CPJ.

[28] Doucette W.R., Schommer J.C. (2018). Pharmacy Technicians’ Willingness to Perform Emerging Tasks in Community Practice. Pharmacy.

[29] Allan M., (Director, Wales Centre for Pharmacy Professional Education) Letter to: Community Pharmacy Wales. 1 leaf. http://www.cpwales.org.uk/getattachment/Services-and-commissioning/Workforce-Development/Pharmacy-Technician-training/PRPT-Letter-to-CPW-Aug18.pdf.aspx?lang=en-GB.

[30] General Pharmaceutical Council Approved Pharmacy Technician Courses 2019. https://www.pharmacyregulation.org/education/pharmacy-technician/accredited-courses.

[31] General Pharmaceutical Council (2017). Standards for the Initial Education and Training of Pharmacy Technicians. https://www.pharmacyregulation.org/sites/default/files/standards_for_the_initial_education_and_training_of_pharmacy_technicians_october_2017.pdf.

[32] General Pharmaceutical Council (2018). Guidance on Tutoring and Supervising Pharmacy Professionals in Training. https://www.pharmacyregulation.org/sites/default/files/document/guidance_on_supervising_pharmacy_professionals_in_training_august_2018.pdf.

[33] Alderman A.K., Salem B. (2010). Survey Research. Plast. Reconstr. Surg..

[34] O’Reilly-Shah V.N. (2017). Factors influencing healthcare provider respondent fatigue answering a globally administered in-app survey. Peer J..

[35] Hughes M., Greenfield T. (2002). Interviewing. Research Methods for Postgraduate.

[36] Block E.S., Erskine L. (2012). Interviewing by Telephone: Specific Considerations, Opportunities and Challenges. Int. J. Qual. Methods.

