# Supplementary material for: Pharmacy Technicians’ Roles and Responsibilities in the Community Pharmacy Sector: A Welsh Perspective
Source: Pharmacy (Basel). 2020 Jun 4;8(2):97. doi: 10.3390/pharmacy8020097 (PMC7356290; doi:10.3390/pharmacy8020097)
Supplement: Supplementary file 1 [file pharmacy-08-00097-s001.pdf]

Participant Interview Schedule

Pre-interview brief:

- Re-confirm background to the project
- Confirm that participant has read the participant information
- Confirm receipt of written consent via e-mail
- Explain how data will be recorded—audio recorded and associated transcripts
- Explain that data will be anonymised using a unique pseudonym
- Explain that audio data and associated transcripts will be stored in a password protected folder and consent forms will be stored in a separate password protected folder
- Confirm that participant can withdraw their interview data from the study at any time
- Check if participant has any questions about the interview process before we begin

Interview schedule:

Please start by telling me about your current role

- What are the core functions of your role?
- Do you undertake any 'extended' roles, e.g. ACPT?
- Does your job description accurately reflect your actual role?
- Has your role changed since registering with the GPhC?
- Has your view of your role changed since registering with the GPhC?

Please describe how your knowledge and skills are used in the workplace.

- Do you feel that your knowledge and skills are utilised to their full extent?

  If the answer is no:
- How else do you think your knowledge and skills could be utilised?
- What are the barriers to you fully utilising your knowledge and skills?

  If the answer is yes:
- Please could you describe how and/or give an example?
- What has enabled you to utilise your knowledge and skills fully?

- Are you qualified/accredited for any roles which you do not have?

To what extent do you feel that your initial training (BTEC +/-NVQ) enabled you to undertake your pharmacy technician role effectively on 'day one'?

- Skill development—professional skills (e.g. problem solving), softer skills (e.g. communication)
- Knowledge and understanding—level, relevance to role

Please describe any further training that you have undertaken.

- Did the further training enable you to undertake your current role effectively, e.g. skill development at a higher level or knowledge and understanding at a higher level?
- Did the training improve your confidence?
- Did the training help you to understand your professional responsibilities in relation to the role, e.g. ACPT?
- Was training/certification necessary for the job role and/or compliance with company policy?
- Are you qualified/accredited for all the roles that you undertake?

Can you tell me about any support or guidance that you receive in relation to your pharmacy technician role?

- Who supports/guides you when there are problems or complaints?
- Who would you go to for advice on professional issues, e.g. reporting a concern?
- Are you able to share any specific examples?

Can you tell me about any factors which you feel have helped you to develop your role?

Can you tell me about any factors which have been barriers to you developing your role?

Who else do you work with and what are their roles?

- Can you describe the difference between the Level 2 Pharmacy Assistant role and Level 3 Pharmacy Technician role in your workplace?
- Do you feel that any of your existing roles could be undertaken by other colleagues just as effectively?
- Who is able to delegate work to others?

Participant Interview De-brief

- Would you like to raise any other issues or make any further comments about the topics that we have discussed today?
- Do you have any other questions about your participation in the interview?
- Thank you very much for your participation
- *Signpost if necessary

*If a participant discloses any concerns which fall outside the remit of this study, then I will advise them to contact one of the following organisations for further guidance:

Association of Pharmacy Technicians UK (professional body)

Address: Association of Pharmacy Technicians UK, One Victoria Square, Birmingham, B1 1BD

Telephone: 0121 632 2025 (voicemail)

General Pharmaceutical Council (regulator)

Address: General Pharmaceutical Council, 25 Canada Square, London, E14 5LQ

Telephone: 0203 713 8000

E-mail: info@pharmacyregulation.org

Online: direct to online Reporting a Concern form

Unison (trade union)

Address: Unison Cymru/Wales, Unison House, Custom House Street, Cardiff, CF10 1AP

Telephone: 0800 0 857 857

E-mail: cymruwales@unison.co.uk
